# Pandemic (H1N1) 2009–associated Deaths Detected by Unexplained Death and Medical Examiner Surveillance

**DOI:** 10.3201/eid1708.101914

**Published:** 2011-08

**Authors:** Christine H. Lees, Catherine Avery, Ryan Asherin, Jean Rainbow, Richard Danila, Chad Smelser, Ann Schmitz, Stephen Ladd-Wilson, Kurt B. Nolte, Kayla Nagle, Ruth Lynfield

**Affiliations:** Author affiliations: Minnesota Department of Health, St. Paul, Minnesota, USA (C.H. Lees, J. Rainbow, R. Danila, K. Nagle, R. Lynfield);; New Mexico Department of Health, Santa Fe, New Mexico, USA (C. Avery, C. Smelser);; Oregon Public Health Department, Portland, Oregon, USA (R. Asherin, S. Ladd-Wilson);; Centers for Disease Control and Prevention, Atlanta, Georgia, USA (A. Schmitz);; University of New Mexico School of Medicine, Albuquerque, New Mexico, USA (K.B. Nolte)

**Keywords:** Influenza A virus, H1N1, influenza, pandemic (H1N1) 2009, Minnesota, Oregon, New Mexico, unexplained deaths, coroners, medical examiners, death, fatal outcome, cause of death, mortality, surveillance, viruses, dispatch

## Abstract

During the pandemic (H1N1) 2009 outbreak, Minnesota, New Mexico, and Oregon used several surveillance methods to detect associated deaths. Surveillance using unexplained death and medical examiner data allowed for detection of 34 (18%) pandemic (H1N1) 2009–associated deaths that were not detected by hospital-based surveillance.

The emergence of pandemic (H1N1) 2009 influenza illustrated the need for improved surveillance to identify deaths resulting from emerging pathogens. Common methods for identifying infectious cause–related deaths include reports by health care providers and review of death certificates. These methods have limitations for identifying deaths caused by emerging pathogens because the disease may not be fully defined or death certificates may not indicate an infectious cause. During an emerging pathogen epidemic, it is important to investigate deaths occurring outside of traditional settings to determine if sudden deaths occurring in the community are a result of the novel pathogen.

In 1995, the Centers for Disease Control and Prevention (CDC) Emerging Infections Program (EIP) Unexplained Deaths Program (UNEX) began in 4 states (1). Under UNEX, deaths likely resulting from an infection, but for which routine testing did not identify a pathogen, are investigated. State and CDC Infectious Diseases Pathology Branch researchers partner with medical examiners and hospital pathologists to review cases and autopsy reports. Expanded resources for specimen testing are provided, which increases the likelihood of a pathogen-specific diagnosis.

The Medical Examiner Infectious Disease Death Surveillance Program (Med-X) was developed in 1999 by the New Mexico Office of the Medical Investigator, New Mexico Department of Health, and Infectious Diseases Pathology Branch to review deaths for infectious causes on the basis of preestablished sets of symptoms and pathologic syndromes (2,3). If there is evidence of an infectious process, specimens are tested to achieve an organism-specific diagnosis. Both UNEX and Med-X have been shown to be useful for bioterrorism and infectious death surveillance (4–6).

The EIP has also established population-based active surveillance for all laboratory-confirmed influenza-related hospitalizations and deaths. Minnesota, New Mexico, and Oregon participate in the UNEX, Med-X, and EIP Influenza Surveillance programs to identify all potential influenza-associated deaths.

## The Study

**Figure 1:**
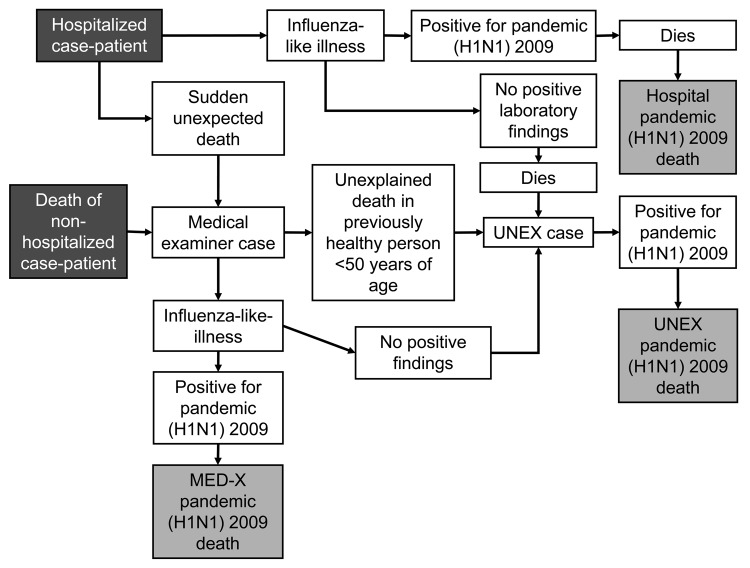
Procedure for evaluating pandemic (H1N1) 2009–associated deaths in Minnesota, New Mexico, and Oregon, April–December 2009. UNEX, Centers for Disease Control and Prevention Emerging Infections Program Unexplained Deaths Program; Med-X, Medical Examiner Infectious Disease Death Surveillance Program.

During the spread of pandemic (H1N1) 2009, UNEX cases were reported to the Minnesota and Oregon health departments by physicians, infection preventionists, and hospital pathologists (Figure 1). Both states also conducted statewide surveillance by using Med-X. New Mexico detected cases through the New Mexico Office of the Medical Investigator and its Med-X system. Medical examiners investigated all decedents for influenza-like illness (ILI) based on pre- or postmortem findings as well as sudden deaths in previously healthy persons <50 years of age. Each state expanded its EIP Influenza Surveillance statewide during the pandemic (H1N1) 2009 pandemic. In addition, hospitalized persons with ILI, including decedents, were reported to the state health department by physicians, infection preventionists, and hospital pathologists.

Pre- and/or postmortem specimens, including nasopharyngeal, nasal, or throat swabs; nasal or endotracheal aspirates; bronchial alveolar lavage specimens; sputum; frozen and fixed respiratory tissue; and serum specimens, were tested at state laboratories or at CDC for pandemic (H1N1) 2009 virus. Tests included PCR, virologic culture, immunohistochemistry, serology, and influenza antigen detection. In a few instances, it was not possible to characterize the virus beyond influenza type A because of limited specimen availability; these cases were assumed to be pandemic (H1N1) 2009. Because UNEX and Med-X are not mutually exclusive, all pandemic (H1N1) 2009–associated deaths were determined to be UNEX/Med-X cases if they were captured through either of those programs (Figure 1).

Data were collected on underlying medical conditions, symptoms, and clinical outcomes from medical records, case investigations, and autopsy reports. In Minnesota and New Mexico, all decedents with positive laboratory findings were reviewed to determine if their deaths were due entirely or in part to pandemic (H1N1) 2009. If influenza was determined not to be related to the death, it was not included as a pandemic (H1N1) 2009–associated death; 7 decedents in Minnesota and 2 in New Mexico were thus excluded. Oregon included all hospital surveillance deaths with positive influenza (H1N1) test results as subtype H1N1 associated without further review, but UNEX/Med-X cases were reviewed for a causal relationship to pandemic (H1N1) 2009. Deaths occurring during April–December 2009 were included in this analysis.

Characteristics of UNEX/Med-X cases versus hospital surveillance cases were compared by using the χ^2^ or Fisher exact test. The Wilcoxon Mann-Whitney test was used to compare medians. SAS version 9.1 software (SAS Institute Inc., Cary, NC, USA) was used for all analyses.

A total of 194 pandemic (H1N1) 2009–associated deaths were detected in this analysis, 160 (82%) through hospital surveillance and 34 (18%) through UNEX/Med-X. The additional surveillance resulted in the detection of 21% more total cases than hospital surveillance alone. Minnesota had the highest proportion of UNEX/Med-X–detected cases with 24% (16/66); Oregon had the lowest with 11% (8/76) (Table 1). Decedents detected by using UNEX/Med-X were more frequently of a nonwhite race (47% vs. 23%); an increased percentage of deaths of American Indians/Alaska Natives was detected through UNEX/Med-X versus hospital surveillance (21% vs. 4%).

**Table 1:** Descriptive characteristics of pandemic (H1N1) 2009–associated deaths, by surveillance program, Minnesota, New Mexico, and Oregon, April–December 2009

Characteristic	Hospital surveillance decedents, n = 160	UNEX/Med-X decedents, n = 34	p value†
State			
Minnesota	50 (76)	16 (24)	
New Mexico	42 (81)	10 (19)	
Oregon	68 (89)	8 (11)	0.09‡
Influenza type§			
Pandemic (H1N1) 2009	82 (89)	25 (96)	
Influenza A, not subtyped	10 (11)	1 (4)	0.45‡
Age, y			
Median	51.0	37.5	<0.001¶
Mean	50.4	33.4	<0.001#
Male gender	94 (59)	17 (50)	0.35
Race/ethnicity			
White	123 (77)	18 (53)	
Black	7 (4)	0 (0)	
American Indian/Alaska Native	7 (4)	7 (21)	
Asian/PacificIslander	2 (1)	2 (6)	
Hispanic	21 (13)	7 (21)	0.001‡
Autopsy performed	27 (17)	29 (85)	<0.001
Place of death			
Hospital/emergency department	146 (91)	15 (44)	
Residence	12 (8)	18 (53)	
Other	1 (1)	0 (0)	
Unknown	1 (1)	1 (3)	<0.001‡

*Values are no. (%) except as indicated. UNEX, Centers for Disease Control and Prevention Emerging Infections Program Unexplained Deaths Program; Med-X, Medical Examiner Infectious Disease Death Surveillance Program. †By χ^2^ or Fisher exact test. ‡By χ^2^ test among all categories. §Data available from Minnesota and New Mexico only; n = 92 for hospital surveillance and n = 26 for UNEX/Med-X. ¶By Wilcoxon Mann-Whitney test. #By analysis of variance F-test.

**Figure 2:**
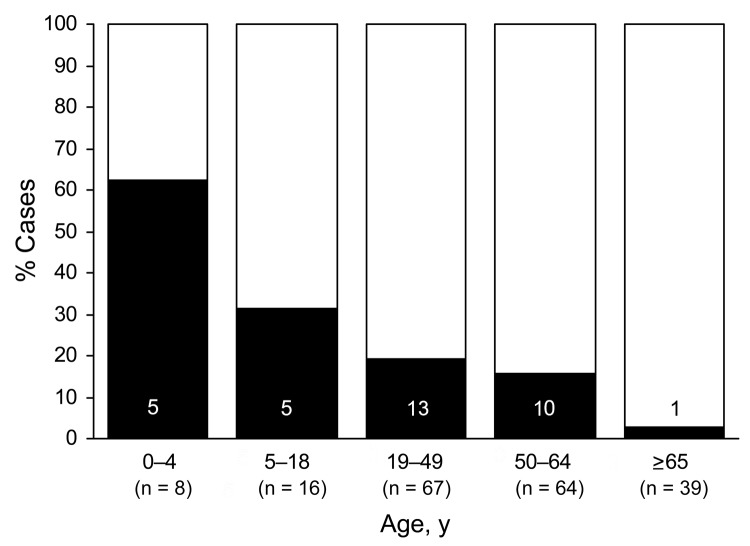
Pandemic (H1N1) 2009–associated deaths, by age group and surveillance program, Minnesota, New Mexico, and Oregon, April–December 2009. White bar sections, deaths detected through hospital surveillance; black bar sections, deaths detected through Centers for Disease Control and Prevention Emerging Infections Program Unexplained Deaths Program and Medical Examiner Infectious Disease Death Surveillance Program.

UNEX/Med-X decedents were more likely to have had an autopsy performed (85% vs. 17%) and were more likely to have died in their residences (53% vs. 8%) than decedents detected by hospital surveillance. The median age of UNEX/Med-X decedents was 37.5 years, compared with 51.0 years for hospital surveillance decedents (p<0.001) (Table 1). The percentage of UNEX/Med-X decedents among age groups decreased with increasing age (62.5% among those 0–4 years of age compared with 2.6% among those >65 years of age; Figure 2).

More hospital surveillance than UNEX/Med-X decedents (89% vs. 68%) were determined to have >1 underlying condition. Specific underlying conditions were more frequently identified among hospital surveillance than UNEX/Med-X decedents, except for obesity (Table 2). Pneumonia, including viral pneumonia, was frequently reported among decedents. Acute respiratory distress syndrome was documented for 37% of hospital and 15% of UNEX/Med-X decedents. Two previously healthy children with nasopharyngeal swabs positive for influenza had evidence at autopsy of viral myocarditis.

**Table 2:** Clinical description of patients whose deaths were associated with pandemic (H1N1) 2009, by surveillance program, Minnesota, New Mexico, and Oregon, April–December 2009*

Underlying conditions	Hospital surveillance decedents, n = 160	UNEX/Med-X decedents, n = 34	p value†
>1 conditions	142 (89)	23 (68)	0.002
Asthma	24 (15)	3 (9)	0.43
Chronic lung disease	51 (32)	7 (21)	0.19
Cardiovascular disease	59 (37)	8 (24)	0.14
Chronic metabolic disease	55 (34)	9 (26)	0.37
Renal disease	17 (11)	1 (3)	0.21
Neuromuscular disorder	19 (12)	2 (6)	0.54
Cancer, past 12 months	10 (6)	0 (0)	0.21
Lymphoma/leukemia	9 (6)	0 (0)	0.36
Immunosuppressive conditions	30 (19)	2 (6)	0.08
Pregnancy	0	0	
Obesity‡	29 (18)	9 (27)	0.27
Morbidly obese‡	23 (14)	4 (12)	1.0
Body mass index‡			
Median	29.2	31.8	0.95§
Mean	32.6	30.7	
Clinical outcomes¶			
Pneumonia	72 (78)	15 (58)	0.04
Viral	12 (17)	6 (40)	
Bacterial#	8 (11)	2 (13)	
Both	8 (11)	1 (7)	
Unknown	44 (61)	6 (40)	
Acute respiratory distress syndrome	34 (37)	4 (15)	0.06
Myocarditis	0 (0)	2 (8)	0.05

*Values are no. (%) except as indicated. UNEX, Centers for Disease Control and Prevention Emerging Infections Program Unexplained Deaths Program; Med-X, Medical Examiner Infectious Disease Death Surveillance Program. †By χ^2^ or Fisher exact test. ‡Obese was defined as either documentation in the medical record of “obese” or a body mass index (BMI) >30 kg/m^2^ and <40 kg/m^2^. Morbidly obese was defined as either documentation in the medical record of “morbidly obese” or a BMI >40 kg/m^2^. If there was a discrepancy, BMI was used. BMI data were available for 80 case-patients and were calculated for adults by using National Institute of Health BMI calculation tables and for children 2–19 years of age by using the Centers for Disease Control and Prevention pediatric BMI calculation. §By Wilcoxon Mann-Whitney test. ¶Data available from Minnesota and New Mexico only; n = 92 for hospital surveillance and n = 26 for UNEX/Med-X. #Bacterial species included 4 *Streptococcus pneumoniae*, 4 methicillin-resistant *Staphylococcus aureus*, 3 methicillin-susceptible *S. aureus*, 2 group A *Streptococcus* spp., and 2 others. Some case-patients had >1 species identified.

## Conclusions

UNEX/Med-X surveillance captured 11%–24% of pandemic (H1N1) 2009–associated deaths in the 3 states. Other estimates of deaths resulting from pandemic (H1N1) 2009 may be increased with better data on nonhospitalized and sudden unexplained deaths (7,8). Estimates from surveillance in New York, New York, which included medical examiner and unexplained respiratory cause–related death surveillance, indicate 17% of decedents died at home and 6% had not sought any prior medical care (9–11).

UNEX/Med-X decedents were younger and more often previously healthy than hospital surveillance decedents, a finding that would change the estimated impact of pandemic (H1N1) 2009 among those populations in particular. Consistent with other studies (12), larger racial/ethnic disparities, particularly among Native American/Alaska Native populations, may be detected by UNEX/Med-X than have been detected through other surveillance methods. Although we were unable to determine the cause of these disparities, the findings warrant further study and attention to these populations regarding public health resources.

Even with an emphasis on deaths among those <50 years of age, UNEX and Med-X programs are critical for detecting severe illnesses that rapidly progress to death and could otherwise go undetected. Partnering with medical examiners and pathologists to identify infectious cause–related deaths among persons who were previously healthy is important to give a clear picture of the entire mortality spectrum.

Although it is important to accurately measure the impact of a disease, it is perhaps more important to quickly identify new serious disease threats. Approximately one tenth to one quarter of the influenza deaths detected in this study, and particularly those in younger, healthier persons, were not detected by hospital surveillance when influenza awareness was at its peak. This finding argues for surveillance systems like UNEX and Med-X as a means of quickly detecting emerging, severe infectious disease threats. Because pathogens are likely to emerge over broad geographic areas, we recommend a standardized approach to death investigations to fully understand the epidemiologic and clinical features of illness caused by a particular pathogen.
